# Identification of prognostic values defined by copy number variation, mRNA and protein expression of *LANCL2* and *EGFR* in glioblastoma patients

**DOI:** 10.1186/s12967-021-02979-z

**Published:** 2021-08-30

**Authors:** Hua-fu Zhao, Xiu-ming Zhou, Jing Wang, Fan-fan Chen, Chang-peng Wu, Peng-yu Diao, Lin-rong Cai, Lei Chen, Yan-wen Xu, Jing Liu, Zong-yang Li, Wen-lan Liu, Zhong-ping Chen, Guo-dong Huang, Wei-ping Li

**Affiliations:** 1 Department of Neurosurgery, Institute of Translational Medicine, The First Affiliated Hospital of Shenzhen University, Shenzhen Second People’s Hospital, Shenzhen, 518035 China; 2 Epilepsy Center, Guangdong 999 Brain Hospital, Guangzhou, 510510 China; 3 Department of Neurosurgery/Neuro-Oncology, Sun Yat-Sen University Cancer Center, Guangzhou, 510060 China; 4 State Key Laboratory of Oncology in South China, Guangzhou, 510060 China; 5 Collaborative Innovation Center for Cancer Medicine, Guangzhou, 510060 China; 6 Department of Neurosurgery, People’s Hospital of Longhua District, Shenzhen, 518109 China; 7 Department of Pathology, The First Affiliated Hospital of Shenzhen University, Shenzhen Second People’s Hospital, Shenzhen, 518035 China

**Keywords:** Glioblastoma, LANCL2, EGFR, Overall survival, Amplification, Overexpression

## Abstract

**Background:**

Epidermal growth factor receptor (EGFR) and lanthionine synthetase C-like 2 (LanCL2) genes locate in the same amplicon, and co-amplification of *EGFR* and *LANCL2* is frequent in glioblastoma. However, the prognostic value of *LANCL2* and *EGFR* co-amplification, and their mRNA and protein expression in glioblastoma remain unclear yet.

**Methods:**

This study analyzed the prognostic values of the copy number variations (CNVs), mRNA and protein expression of *LANCL2* and *EGFR* in 575 glioblastoma patients in TCGA database and 100 glioblastoma patients in tumor banks of the Shenzhen Second People’s Hospital and the Sun Yat-sen University Cancer Center.

**Results:**

The amplification of *LANCL2* or *EGFR*, and their co-amplification were frequent in glioblastoma of TCGA database and our tumor banks. A significant correlation was found between the CNVs of *LANCL2* and *EGFR* (*p* < 0.001). CNVs of *LANCL2* or *EGFR* were significantly correlated with *IDH1/2* mutation but not *MGMT* promoter methylation. Multivariate analysis showed that *LANCL2* amplification was significantly correlated with reduced overall survival (OS) in younger (< 60 years) glioblastoma patients of TCGA database (*p* = 0.043, HR = 1.657) and our tumor banks (*p* = 0.018, HR = 2.199). However, *LANCL2* or *EGFR* amplification, and their co-amplification had no significant impact on OS in older (≥ 60 years) or *IDH1/2*-wild-type glioblastoma patients. mRNA and protein overexpression of LANCL2 and EGFR was also frequently found in glioblastoma. The mRNA expression rather than the protein expression of *LANCL2* and *EGFR* was positively correlated (*p* < 0.001). However, mRNA or protein expression of EGFR and LANCL2 was not significantly correlated with OS of glioblastoma patients. The protein expression level of LANCL2, rather than EGFR, was elevated in relapsing glioblastoma, compared with newly diagnosed glioblastoma. In addition, the intracellular localization of LanCL2, not EGFR, was associated with the grade of gliomas.

**Conclusions:**

Taken together, amplification and mRNA overexpression of *LANCL2* and *EGFR*, and their co-amplification and co-expression were frequent in glioblastoma patients. Our findings suggest that amplification of *LANCL2* and *EGFR* were the independent diagnostic biomarkers for glioblastoma patients, and *LANCL2* amplification was a significant prognostic factor for OS in younger glioblastoma patients.

**Supplementary Information:**

The online version contains supplementary material available at 10.1186/s12967-021-02979-z.

## Background

Glioblastoma multiforme (glioblastoma, GBM), belonging to the highest World Health Organization (WHO) grade glioma (grade IV), is the most common malignant and aggressive primary brain tumor (47.7%) with a high mortality rate [1, 2]. According to the 2018 CBTRUS report, the incidence rate of GBM is 3.21 per 100,000 populations, which is the highest in malignant brain tumors [2, 3]. Under the Stupp’s therapeutic protocol (maximal surgical resection followed by adjuvant radiotherapy and chemotherapy with temozolomide), the median overall survival of GBM patients is 14.6 months, while the 2-year overall survival (OS) rate and progression-free survival (PFS) rate is 26.5% and 10.7%, respectively [4]. Aberrations of molecular markers such as O(6)-methylguanine-DNA methyltransferase (*MGMT*) promoter methylation, codeletion of 1p and 19q, isocitrate dehydrogenase 1 and 2 (*IDH1* and *IDH2*) mutation, telomerase reverse transcriptase (*TERT)* promoter mutation, *TP53* mutation, and epidermal growth factor receptor (EGFR) overexpression show prognostic significance to guide treatment decisions of GBM patients. In particular, *IDH1/2* gene mutations are found in more than 70% of grade II-III glioma and secondary GBM that arises from low-grade glioma. GBM patients with *IDH1/2* gene mutations often have a better clinical outcome than those with wild-type *IDH* [5]. *TERT* promoter mutation is found in approximately 80% of patients with primary GBM that develops rapidly without any clinical or histologic evidence of a less malignant precursor lesion. GBM patients with *TERT* promoter mutation often have poor survival and a high risk of death [6].

EGFR, a member of receptor tyrosine kinases (RTKs), is essential to the pathological process in various cancers via activation of PI3K/Akt signaling pathway. Analyzed by The Cancer Genome Atlas (TCGA) database, overall alterations including amplification, mutation, rearrangement and altered splicing of *EGFR* gene in GBM are highly frequent (57.4%) [7]. Compared with secondary GBM, EGFR amplification (36%) and overexpression (more than 60%) are more common in primary GBM [8, 9]. Evidence shows that *EGFR* gene amplification has a strong correlation with EGFR overexpression. Approximate 98% of primary GBM with *EGFR* amplification also exhibit EGFR overexpression, while 70%-90% of those with EGFR overexpression show *EGFR* amplification [8, 10]. A number of studies demonstrate that the amplification and overexpression of EGFR are associated with poor prognosis of GBM patients, especially young people [10–12]. However, a retrospective study shows that *EGFR* amplification is not a prognostic factor for GBM patients treated with surgery. And a meta-analysis also shows that *EGFR* amplification is not significantly associated with OS of GBM patients, indicating a heterogeneity of significance among difference studies and subjects [13].

Lanthionine synthetase C-like 2 (LanC Like 2, LanCL2), a member of eukaryotic LanC-like protein family, is a homologue of prokaryotic LanC involved in the synthesis of the antibiotic named as lantibiotics [14]. LanCL2 is a receptor of abscisic acid (ABA) which is not only a plant hormone but also an endogenous mammalian hormone involved in glycemic control [15]. It is also known as testis adriamycin sensitivity protein (TASP) that is able to increase sensitivity of tumor cells to adriamycin via reduction of P-glycoprotein [16]. Accumulating evidence show that LanCL2 plays important roles in the regulation of stress response, inflammation and glycometabolism, providing a potential target for the treatment of chronic inflammatory, metabolic and immune-related diseases [17, 18]. *LANCL2*, along with *SEC61G* and *ECOP* genes, are located in the flank of *EGFR* gene at chromosomal 7p11.2. These genes are in the same amplicon, and their co-amplification with *EGFR* is common in GBM patients [19, 20]. However, it is not clear that whether co-amplification of *EGFR* and *LANCL2* has prognostic value for GBM patients, and what are their mRNA and protein expression patterns.

Here, this study analyzed the copy number variations (CNVs), mRNA and protein expression profiles, and their prognostic values of *LANCL2* and *EGFR* in GBM specimens from TCGA database or from the tumor banks of Shenzhen Second People’s Hospital and Sun Yat-sen University Cancer Center. We showed that amplification and mRNA overexpression of *LANCL2* and *EGFR*, and their co-amplification and co-expression were frequent in GBM patients. Amplification of *LANCL2* and *EGFR* were the independent diagnostic biomarkers for GBM patients, and *LANCL2* amplification was a significant prognostic factor for OS in younger GBM patients. The protein expression pattern and role of LanCL2 were independent to EGFR. LanCL2 overexpression was correlated with glioblastoma recurrence, and its activation may trigger its translocation into the nucleus.

## Methods

### TCGA database analysis

CNVs and mRNA expression data analyzed using the GISTIC2 algorithm in the TCGA database were achieved in the cBio Cancer Genomics Portal (http://www.cbioportal.org) [21, 22]. The TCGA Pan-Cancer Atlas dataset involving more than 11,000 human tumors across 33 different cancer types was selected [23–26]. The clinical data of GBM from the TCGA Pan-Cancer database were downloaded to analyze the OS and PFS of the cohort using Kaplan–Meier survival analysis and log-rank test. Chi-square test was carried out to estimate the correlation of CNVs, while the correlation of mRNA expression (RNASeq V2 RSEM) were calculated by Pearson’s correlation. CNVs including shallow deletion (possibly heterozygous deletion), diploid, low-level gain and high-level amplification were defined as the putative copy number values of − 1, 0, 1 and 2, respectively.

### Tumor specimens

Tumor specimens were retrospectively obtained from the tumor banks in the Shenzhen Second People’s Hospital and the Sun Yat-sen University Cancer Center. All tumor samples were histologically diagnosed as GBM (WHO grade IV). Four human normal brain tissues (including two craniocerebral trauma, one para-carcinoma and one epilepsy) and four grade I gliomas were used as the negative controls. Identification of all tumor samples or normal brain tissues were confirmed by an experienced pathologist. This study was approved by the Research Ethics Committee of Shenzhen Second People’s Hospital and Sun Yat-sen University Cancer Center. All patients were given written informed consent.

### DNA extraction and copy number assay

Genomic DNA (gDNA) was extracted using QIAamp DNA Mini Kit (QIAGEN) and copy number variations were evaluated by TaqMan Copy Number Assays (Thermo Scientific) following the manufacturer’s instructions. The Taqman Copy Number Assay probes for *LANCL2* (Hs04953915_cn) and *EGFR* (Hs04983302_cn) genes were used for copy number quantitation, while TaqMan Copy Number Reference Assay RNase P was served as the reference. TaqMan Genotyping Master Mix was employed for the PCR amplification procedure, and 20 ng/well of gDNA was added in each PCR reaction, which was performed in ABI Quantstudio™ DX. Each reaction was duplicated.

### Western blotting

Total proteins were extracted by RIPA lysis buffer and protein concentrations were determined using the BCA protein assay (Thermo Scientific). Proteins were then separated by 8% SDS-PAGE and transferred to PVDF membranes (Millipore). After blocking with 5% non-fat milk or 5% BSA, membranes were incubated with gentle agitation in primary antibodies (1:1000) overnight at 4 °C and then in HRP-conjugated secondary antibodies (1:5000) for 1 h at room temperature. Positive signals were visualized by ECL chemiluminescence using ChemiDoc MP Imaging System (Bio-Rad).

### Immunohistochemistry (IHC)

Tissue microarray slides containing 60 to 80 of paraffin-embedded glioma tissue specimens (Cat.No: HBra-Gli060PG-01 and HBra-G080PG-01) were purchased from Shanghai Outdo Biotech Company. Slide HBra-Gli060PG-01 included 3 normal brain tissues (1 white matter and 2 cortex), 3 grade I, 9 grade II, 9 grade III, and 34 grade IV gliomas (GBM). Slide HBra-G080PG-01 included 3 normal brain tissues (1 white matter and 2 cortex), 3 grade I, 8 grade II, 22 grade III, and 44 grade IV gliomas (GBM), which shared 54 samples with HBra-Gli060PG-01. The use of human tissues in tissue microarray slides was approved by the Ethics Committee of Shanghai Outdo Biotech Company. Sections were immunostained with appropriate primary antibody and biotin-conjugated goat anti-rabbit IgG. After the detection using DAB detection kit (Boster), slides were counterstained with hematoxylin, dehydrated and mounted. IHC staining scores were calculated as the product of the proportion of positive staining cells (0–4) and the intensity of staining (0–3). The proportion of positive staining cells was graded as followed: 0 (no staining); 1 (1%–25%, including 25%); 2 (25%–50%, including 50%); 3 (50%–75%, including 75%); 4 (> 75%). The intensity of staining was graded as followed: negative = 0; weakly positive = 1; positive = 2; strongly positive = 3.

### Statistical analysis

Data were presented as mean ± S.E.M and all statistical analyses were carried out using GraphPad Prism 8 and SPSS Statistic 22.0 software. Relative protein expression was evaluated by measurement of density of Western blotting bands using Image J software. Difference among groups which did not follow a normal distribution was compared using the Mann–Whitney U test or Kruskal–Wallis One-way ANOVA with Dunn's multiple comparisons test. Patients’ survival analyzed using the Kaplan–Meier method and the log-rank test was used for univariate analysis. Multivariate analysis of OS was performed using the Cox proportional hazards regression model in a forward stepwise manner. The distribution of categorical values within two groups was analyzed by the chi-square test (Fisher’s exact test). The difference was considered to be significant at *p* < 0.05. The licenses of software are available under any requirement for permission for use.

## Results

### Amplification and co-amplification of *LANCL2* and *EGFR* were prevalent in glioblastoma, and *LANCL2* amplification was an independent prognostic factor for younger glioblastoma patients

**Fig. 1:**
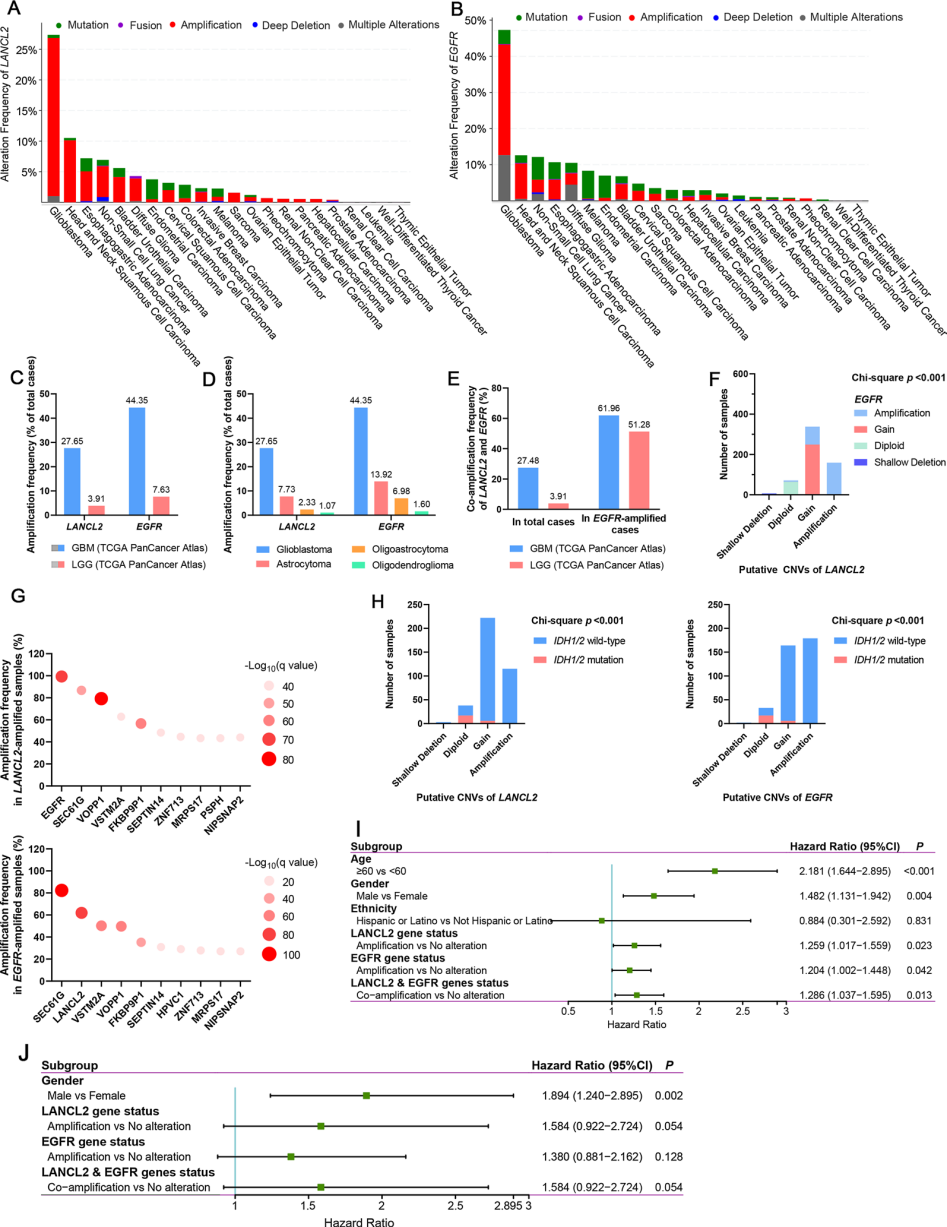
Amplification and co-amplification of *LANCL2* and *EGFR* were frequent in GBM specimens of TCGA database, and *LANCL2* amplification were associated with poor OS in younger GBM patients. **A**, **B** Genetic alteration frequencies of *LANCL2* and *EGFR* in 32 different cancers of TCGA Pan-Cancer Atlas database. The diagrams showed the top 22 cancers with the highest frequencies. **C** Amplification frequencies of *LANCL2* and *EGFR* in two TCGA studies of Glioblastoma Multiforme (n = 592) and Brain Lower Grade Glioma (n = 514). **D** Amplification frequencies of *LANCL2* and *EGFR* in different pathological types of gliomas. **E** Co-amplification frequencies of *LANCL2* and *EGFR* in total or *EGFR*-amplified GBM samples. **F** Chi-square test (Fisher’s exact test) showed the CNVs of *LANCL2* and *EGFR* in GBM were significantly associated. **G** The top 10 genes with the highest amplification frequencies in *LANCL2*- or *EGFR*-amplified GBM samples. **H** Chi-square test showed that CNVs of *LANCL2* and *EGFR* were significantly correlated with *IDH1/2* mutation status in GBM. **I** Forest plot showing the univariate analysis for OS in GBM patients of TCGA database. **J** Forest plot showing the univariate analysis for OS in younger (< 60 years) GBM patients of TCGA database. *P* values and hazard ratios were determined by log-rank test

Firstly, to investigate the CNVs of *LANCL2* and *EGFR* genes in a panel of cancers, 32 studies of different cancer types in TCGA Pan-Cancer Atlas database (n = 10,967) were selected. Results showed that the dominant genomic alterations of *LANCL2* and *EGFR* in cancers were amplification and mutation, while gene fusion and deep deletion were rare. Glioblastoma, head and neck squamous cell carcinoma, esophagogastric adenocarcinoma and non-small cell lung cancer were the top four tumors with the highest alteration frequencies of *LANCL2* and *EGFR* (Fig. 1A, B). Subsequently, two studies Glioblastoma Multiforme (n = 592) and Brain Lower Grade Glioma (n = 514) were further analyzed. The amplification frequencies of *LANCL2* and *EGFR* in GBM were up to 27.65% (159 of 575 cases) and 44.35% (255 of 575 cases), whereas those in low-grade glioma (LGG) were only 3.91% (20 of 511 cases) and 7.63% (39 of 511 cases), respectively (Fig. 1C). The data of LGG contained grade II and III gliomas, including oligodendroglioma, oligoastrocytoma and astrocytoma. Among the LGG data, the amplification frequencies of *LANCL2* and *EGFR* in astrocytoma were the highest (7.33% and 13.92%, respectively), while those in oligoastrocytoma were the lowest (1.07% and 1.60%, respectively) (Fig. 1D). Co-amplification of *LANCL2* and *EGFR* was common in GBM, but it was rare in LGG. *LANCL2* amplification was found in 61.96% of GBM samples and 51.28% of LGG samples containing *EGFR* amplification. Furthermore, nearly all GBM and LGG samples containing *LANCL2* amplification displayed *EGFR* amplification (Fig. 1E). The main types of *LANCL2* and *EGFR* CNVs in GBM were copy number gain and amplification, whereas shallow deletion and diploid were infrequent. Chi-square test demonstrated a significant correlation between the CNVs of *LANCL2* and *EGFR* (*p* < 0.001) (Fig. 1F). We analyzed the top ten genes which had the highest co-amplification frequencies with *LANCL2* or *EGFR*. Results indicated that the amplification frequencies of *EGFR*, *SEC61G* and *VOPP1* genes were the top three highest in *LANCL2*-amplified GBM samples, while *SEC61G*, *LANCL2* and *VSTM2A* were the top three genes co-amplified with *EGFR* (Fig. 1G). The relationship between CNVs of *LANCL2*/*EGFR* and molecular pathology of GBM samples was analyzed. Wild-type *IDH1/2* was mainly found in GBM samples with *LANCL2/EGFR* gain or amplification. Chi-square test found that CNVs of *LANCL2* or *EGFR* were significantly correlated with *IDH1/2* mutation but not *MGMT* methylation status (Fig. 1H, Additional file 1: Figure S1A, B).

Univariate analysis was performed to evaluate the difference of OS and PFS for the variables (age, gender, ethnicity, *LANCL2* and *EGFR* genes status). Results showed that old age (≥ 60 yrs), gender (male), *LANCL2* or *EGFR* amplification and their co-amplification were the significant factors contributing to shorter OS, whereas age was the only variable markedly associated with PFS (Table 1). Hazard ratios (HR) of these variables were demonstrated in the forest plot (Fig. 1I). Kaplan–Meier survival curves were also drawn in Additional file 1: Figure S1C. Ethnicity did not have a significant impact on OS, which may be due to the lack of the number of Hispanic or Latino (n = 5). Thus, ethnicity was excluded as a covariate in the subsequent multivariate analysis. Since only 284 GBM patients have all the data of age, gender, *LANCL2* and *EGFR* genes status, we performed multivariate analysis for OS on theses 284 patients of TCGA database. Results showed that age (*p* < 0.001, HR = 2.221) and gender (*p* = 0.029, HR = 1.382) were the independent prognostic factors for OS. The prognostic values of *LANCL2* or *EGFR* amplification and their co-amplification were not sufficient to reach significance (Table 2). Further, according to the age at diagnosis, GBM patients were divided into two categories: younger (< 60 yrs) and older (≥ 60 yrs) patients. Univariate analysis found that only gender (*p* = 0.002, HR = 1.894) were significantly correlated with OS of younger GBM patients. *LANCL2* amplification and *LANCL2* & *EGFR* co-amplification displayed the same results of univariate analysis, since all the patients with *LANCL2* amplification had *LANCL2* & *EGFR* co-amplification (Fig. 1J, Table 3). Surprisingly, multivariate analysis showed that gender (*p* = 0.002, HR = 2.029) and *LANCL2* amplification (*p* = 0.043, HR = 1.657) were independent significant prognostic factors for OS in younger GBM patients (Table 4). However, these variables (including gender, *LANCL2* and *EGFR* genes status) had no significant impact on OS in older (≥ 60 yrs) GBM patients (Additional file 1: Table S1). Due to the strong association between *LANCL2/EGFR* amplification and wild-type *IDH1/2*, univariate and multivariate analyses for survival was performed to investigate the prognostic values of *LANCL2* and *EGFR* amplification in *IDH1/2*-wild-type GBM patients. Results found that only age and gender had a significant impact on OS of *IDH1/2*-wild-type GBM patients, whereas *LANCL2* or *EGFR* amplification and their co-amplification were not significantly correlated with OS and PFS of *IDH1/2*-wild-type GBM patients (Additional file 1: Figure S1D, Tables S2, S3).

**Table 1:** Univariate analysis for OS and PFS in GBM patients of TCGA database

Variable	No. (%)	Median OS (months)	*P*	Median PFS (months)	*P*
Age (years)			< 0.001		0.007
≥ 60	152 (52.96)	11.28		5.82	
< 60	135 (47.04)	17.79		7.86	
Gender			0.009		0.145
Male	169 (58.89)	13.35		7.04	
Female	118 (41.11)	15.65		7.36	
Ethnicity			0.954		0.686
Hispanic or Latino	5 (2.21)	14.22		5.98	
Not Hispanic or Latino	221 (97.79)	14.01		7.04	
LANCL2 gene status			0.023		0.123
Amplification	157 (27.12)	13.78		6.67	
No alteration	422 (72.88)	14.50		7.20	
EGFR gene status			0.042		0.230
Amplification	252 (43.52)	14.01		6.84	
No alteration	327 (56.48)	14.50		7.66	
LANCL2 and EGFR genes status			0.013		0.509
Co-amplification	156 (26.94)	13.78		6.67	
No alteration	423 (73.06)	14.53		7.30	
LANCL2 mRNA status			0.224		0.664
Overexpression	56 (36.36)	13.78		7.04	
No alteration	98 (63.64)	13.61		5.98	
EGFR mRNA status			0.778		0.136
Overexpression	75 (48.70)	14.93		6.41	
No alteration	79 (51.30)	12.95		6.90	
LANCL2 and EGFR mRNA status			0.930		0.689
Concurrent overexpression	42 (27.27)	15.39		6.41	
No alteration	112 (72.73)	13.12		6.84

**Table 2:** Multivariate analysis by the Cox proportional hazard regression model for OS in GBM patients of TCGA database

Variable	HR (95% CI)	*P*
Age (years)		
≥ 60 vs < 60	2.221 (1.667–2.961)	< 0.001
Gender		
Male vs female	1.382 (1.034–1.848)	0.029
LANCL2 gene status		
Amplification vs no alteration	NA	0.351
EGFR gene status		
Amplification vs no alteration	NA	0.799
LANCL2 and EGFR genes status		
Co-amplification vs no alteration	NA	0.553

*HR* hazard ratio, *CI* confidence interval, *NA* not applicable

**Table 3:** Univariate analysis for OS in younger GBM patients (age < 60 yrs) of TCGA database

Variable	No.(%)	Median OS (months)	*P*	Median PFS (months)	*P*
Gender			0.002		0.131
Male	78 (57.78)	15.39		7.86	
Female	57 (42.22)	22.49		8.48	
LANCL2 gene status			0.054		0.177
Amplification	33 (24.44)	15.02		5.85	
No alteration	102 (75.56)	18.08		8.48	
EGFR gene status			0.128		0.795
Amplification	56 (41.48)	17.49		8.12	
No alteration	79 (58.52)	17.79		7.63	
LANCL2 and EGFR genes status			0.054		0.177
Co-amplification	33 (24.44)	15.02		5.85	
No alteration	102 (75.56)	18.08		8.48

**Table 4:** Multivariate analysis by the Cox proportional hazard regression model for OS in younger GBM patients (age < 60 yrs) of TCGA database

Variable	HR (95% CI)	*P*
Gender		
Male vs female	2.029 (1.286–3.201)	0.002
LANCL2 gene status		
Amplification vs no alteration	1.657 (1.017–2.699)	0.043
EGFR gene status		
Amplification vs no alteration	NA	0.624
LANCL2 and EGFR gene status		
Co-amplification vs no alteration	NA	NA

*HR* hazard ratio, *CI* confidence interval, *NA* not applicable

### mRNA overexpression of *LANCL2* and *EGFR* were frequent in glioblastoma, but were not associated with the prognosis of glioblastoma patients

**Fig. 2:**
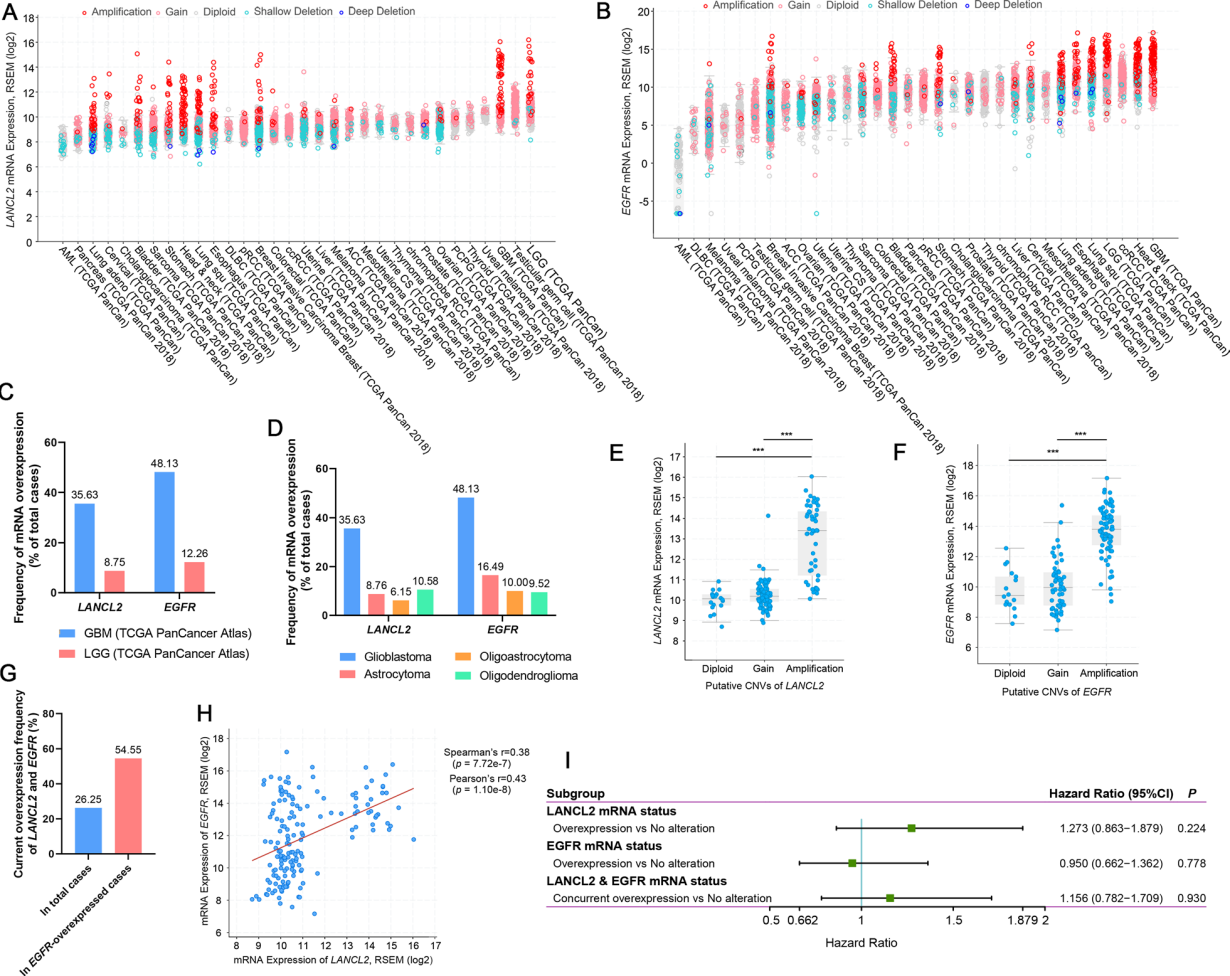
mRNA overexpression of *LANCL2* and *EGFR* was common in GBM specimens of TCGA database, but was not associated with prognosis of GBM patients. **A**, **B** mRNA expression levels of *LANCL2* and *EGFR* in 32 different cancers of TCGA Pan-Cancer Atlas database. **C** Frequencies of *LANCL2* and *EGFR* mRNA overexpression in two TCGA studies of GBM and LGG. **D** Frequencies of *LANCL2* and *EGFR* mRNA overexpression in different pathological types of gliomas. **E**, **F** The correlations between CNVs and mRNA expression of *LANCL2* or *EGFR* in GBM. *P* values were determined by Kruskal–Wallis One-Way ANOVA and Dunn’s multiple comparisons. ***p < 0.001. **G** Frequencies of concurrent mRNA overexpression of *LANCL2* and *EGFR* in total or *EGFR*-overexpressed GBM samples. **H** A significant association between the mRNA expression levels of *LANCL2* and *EGFR* in GBM. **I** Forest plot showing the univariate analysis for OS according to the mRNA expression of *LANCL2* and *EGFR* in GBM patients of TCGA database. *P* values and hazard ratios were determined by log-rank test

The mRNA expression profiles of *LANCL2* and *EGFR* were investigated in 32 different cancers of TCGA database. In the histograms, the average mRNA expression of *LANCL2* and *EGFR* was organized from lowest to highest priority. Among them, LGG, testicular germ cell carcinoma, GBM and uveal melanoma were the top four tumors with the highest average mRNA expression of *LANCL2*, while the average mRNA expression of *EGFR* was highest in GBM, head and neck cancer, clear cell renal cell carcinoma (ccRCC) and LGG (Fig. 2A, B). mRNA overexpression of *LANCL2* and *EGFR* was found in 35.63% (57 of 160 cases) and 48.13% (77 of 160 cases) of GBM samples, respectively (Fig. 2C). However, the mRNA overexpression frequencies of *LANCL2* and *EGFR* in LGG samples were only around 10%, and little difference was shown in astrocytoma, oligoastrocytoma and oligodendroglioma (Fig. 2D). The correlation between mRNA expression and CNV of *LANCL2* and *EGFR* was then analyzed. Results showed that mRNA expression of *LANCL2* was significantly elevated in GBM samples with *LANCL2* amplification, compared with GBM samples with diploid or gain of *LANCL2* (Fig. 2E)*.* Likewise, the correlation was the same in *EGFR* (Fig. 2F). Concurrent mRNA overexpression of *LANCL2* and *EGFR* was found in 26.25% (42 of 160 cases) of total GBM samples and 54.55% (42 of 77 cases) of *EGFR*-overexpressed GBM samples (Fig. 2G). In addition, linear regression analysis demonstrated that mRNA expression of *LANCL2* and *EGFR* was positively correlated (*p* < 0.001)(Fig. 2H). To investigate the prognostic values of *LANCL2* or *EGFR* mRNA expression, Kaplan–Meier survival and univariate analyses were performed. We showed that mRNA overexpression of *LANCL2* or *EGFR*, and their concurrent overexpression were not significantly associated with OS and PFS of GBM patients (Table 1, Fig. 2I, Additional file 1: Figure S2A). Interestingly, mRNA expression levels of *EGFR* were significantly elevated in *IDH1/2*-wild-type GBM samples, while no obvious change of *LANCL2* mRNA expression was found, suggesting a significant association between *EGFR* mRNA expression and *IDH1/2* status (Additional file 1: Figure S2B). However, mRNA overexpression of *LANCL2* or *EGFR* was also not significantly associated with OS and PFS of *IDH1/2*-wild-type GBM patients (Additional file 1: Figure S2C).

### Amplification and co-amplification *LANCL2* and *EGFR* were also frequent in glioblastoma from the tumor banks, and *LANCL2* amplification was associated with poor overall survival of glioblastoma patients

**Fig. 3:**
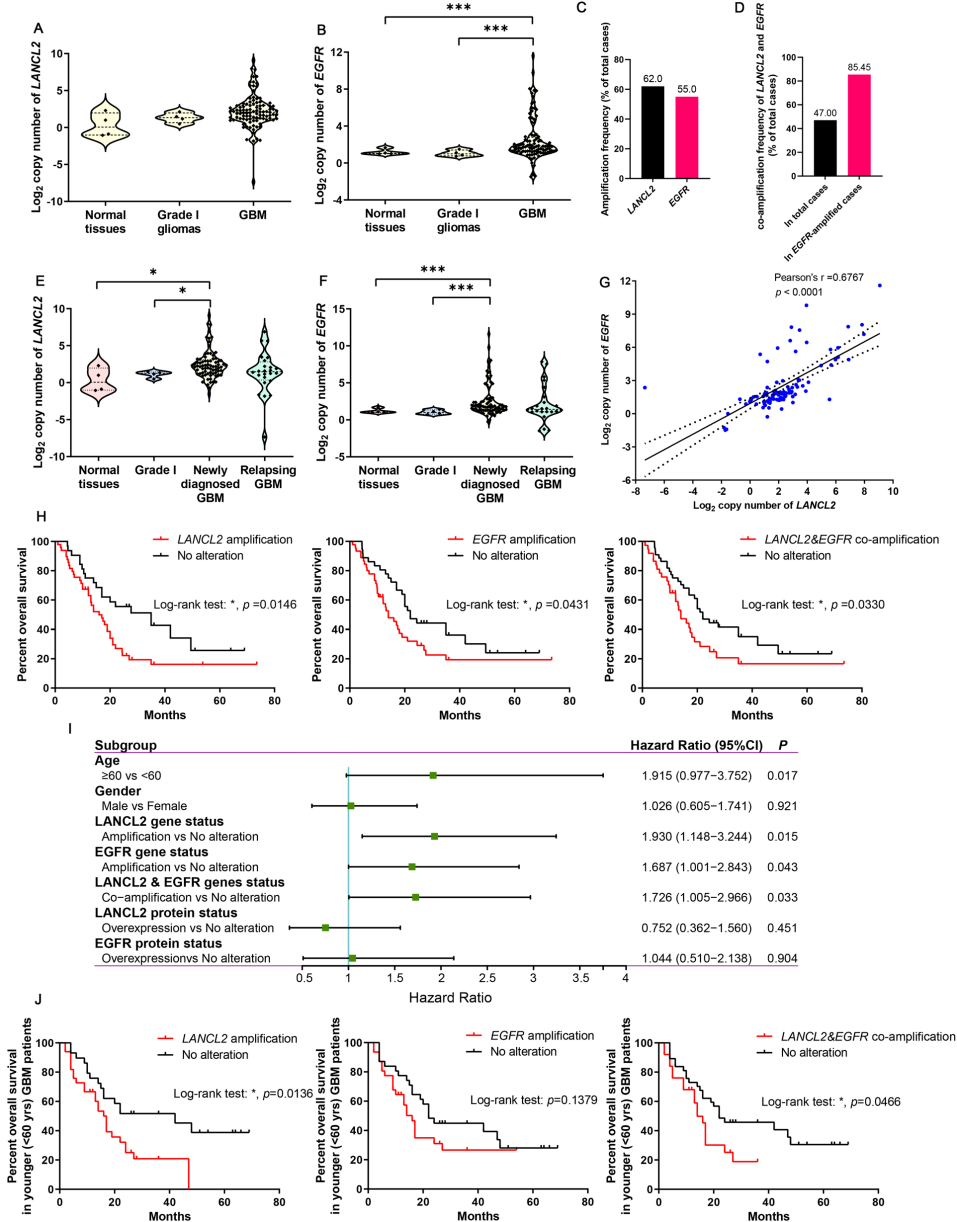
Amplification, co-amplification and their prognostic values of *LANCL2* and *EGFR* were verified in GBM samples of tumor banks. **A**, **B** Copy numbers of *LANCL2* and *EGFR* in GBM samples of our tumor banks. Normal brain tissues and grade I gliomas were used for comparison. *P* values were determined by Kruskal–Wallis One-Way ANOVA and Dunn’s multiple comparisons. ***p < 0.001. **C** Amplification frequencies of *LANCL2* and *EGFR* in GBM samples of tumor banks. **D** Co-amplification frequencies of *LANCL2* and *EGFR* in total or *EGFR*-amplified GBM samples. **E**, **F** Copy numbers of *LANCL2* and *EGFR* in newly diagnosed and relapsing GBM samples. Normal brain tissues and grade I gliomas were used for comparison. *P* values were determined by Kruskal–Wallis One-Way ANOVA and Dunn’s multiple comparisons. *p < 0.05; ***p < 0.001. **G** Pearson’s correlation analysis showed that the copy numbers of *LANCL2* and *EGFR* in GBM were significantly correlated. **H** Kaplan–Meier survival analysis of *LANCL2* or *EGFR* amplification, and their co-amplification for OS in GBM patients (*p < 0.05). **I** Forest plot showing the univariate analysis for OS in GBM patients. *P* values and hazard ratios were determined by log-rank test. **J** Kaplan–Meier survival analysis of *LANCL2* or *EGFR* amplification, and their co-amplification for OS in younger (< 60 years) GBM patients (*p < 0.05)

To validate the analysis results of TCGA database, we analyzed the copy numbers of 100 GBM patients’ samples from our tumor banks by Taqman Copy Number Assay using fluorescent probes targeting *LANCL2* and *EGFR*. The log_2_ copy number value larger than 2 was regarded as amplification. Results showed that compared with the copy numbers in normal brain tissues and grade I gliomas, the copy numbers of *EGFR* were significantly elevated in GBM, while the copy numbers of *LANCL2* had no obvious changes (Fig. 3A, B). Interestingly, when the GBM samples were subdivided into newly diagnosed and relapsing tumors, the copy numbers of *LANCL2* and *EGFR* were significantly increased only in newly diagnosed GBM (Fig. 3E, F). The amplification frequencies of *LANCL2* and *EGFR* were 62.00% and 55.00% in 100 GBM patients, respectively (Fig. 3C, Table 4). *LANCL2* and *EGFR* co-amplification was found in 47.00% of the total GBM samples and 85.45% of GBM samples containing *EGFR* amplification (Fig. 3D, Additional file 1: Table S5). Pearson’s correlation analysis also showed that the copy numbers of *LANCL2* and *EGFR* were positively correlated with each other (Fig. 3G). Kaplan–Meier survival and univariate analyses demonstrated that age, *LANCL2* or *EGFR* amplification, and their co-amplification were significantly associated with decreased OS of GBM patients (n = 81), whereas gender was not a significant variable (Fig. 3H, I). Therefore, gender was excluded as a covariate in the subsequent multivariate analysis, which showed that only *LANCL2* amplification was a significant prognostic factor for OS (*p* = 0.004, HR = 2.319) (Table 5). We subsequently divided GBM patients into younger (< 60 yrs) and older (≥ 60 yrs) groups. Kaplan–Meier survival and multivariate analyses also found that *LANCL2* amplification was significantly associated with poor OS (*p* = 0.018, HR = 2.199) in younger GBM patients (n = 62) (Fig. 3J, Table 6). However, gender, *LANCL2* and *EGFR* amplification had no significant influence on OS of older GBM patients (n = 19) (Additional file 1: Table S4).

**Table 5:** Multivariate analysis by the Cox proportional hazard regression model for OS in GBM patients from tumor banks

Variable	HR (95% CI)	*P*
Age (years)		
≥ 60 vs < 60	NA	0.117
LANCL2 gene status		
Amplification vs no alteration	2.319 (1.306–4.115)	0.004
EGFR gene status		
Amplification vs no alteration	NA	0.605
LANCL2 and EGFR genes status		
Co-amplification vs no alteration	NA	0.656

*HR* hazard ratio, *CI* confidence interval, *NA* not applicable

**Table 6:** Multivariate analysis by the Cox proportional hazard regression model for OS in younger GBM patients (age < 60 years) from tumor banks

Variable	HR (95% CI)	*P*
Gender		
Male vs female	NA	0.791
LANCL2 gene status		
Amplification vs no alteration	2.199 (1.142–4.236)	0.018
EGFR gene status		
Amplification vs no alteration	NA	0.805
LANCL2 and EGFR genes status		
Co-amplification vs no alteration	NA	0.845

*HR* hazard ratio, *CI* confidence interval, *NA* not applicable

Chi-square tests showed that amplification of *LANCL2* was not correlated with *IDH1* and *TERT* mutations, and *MGMT* methylation, whereas amplification of *EGFR* was significantly associated with *IDH1* and *TERT* mutations. On the other side, co-amplification of *LANCL2* and *EGFR* was not related with *TERT* mutation and *MGMT* methylation, but was correlated with *IDH1* mutation (Additional file 1: Figure S3A). Kaplan–Meier survival analysis showed that *LANCL2* or *EGFR* amplification, and their co-amplification were not correlated with OS in *IDH1*-wild-type GBM patients (n = 20) (Additional file 1: Figure S3B).

### Protein expression and localization of LanCL2 was independent to EGFR in gliomas

**Fig. 4:**
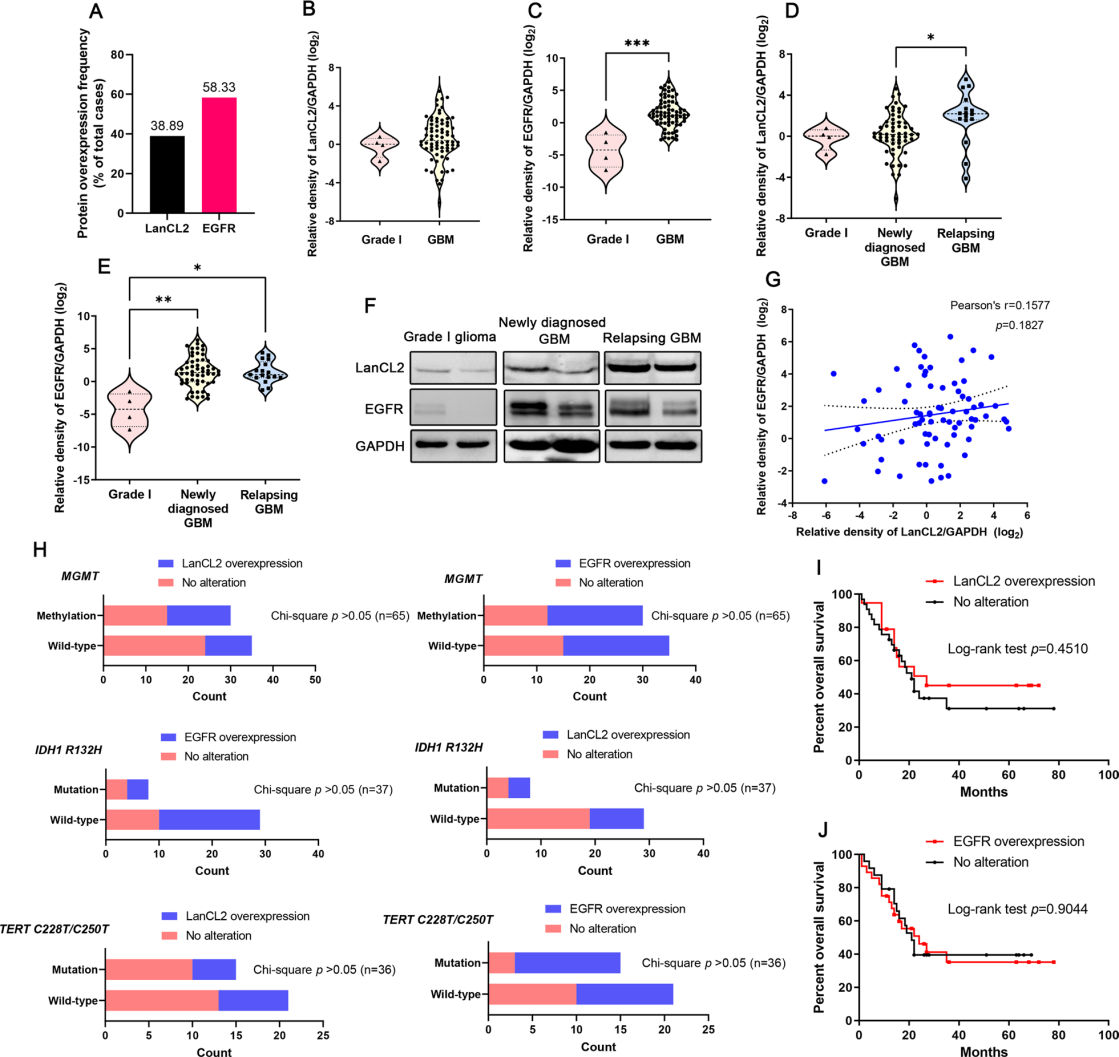
Protein expression profiles and prognostic values of LanCL2 and EGFR in GBM samples of tumor banks. **A** Frequencies of LanCL2 and EGFR protein overexpression in GBM samples of our tumor banks. **B**, **C** Relative expression of LanCL2 and EGFR in GBM samples was evaluated by measurement of density of immunoblotting bands. *P* values were determined by Mann–Whitney U test. ***p < 0.001. **D**, **E** Relative expression of LanCL2 and EGFR in newly diagnosed and relapsing GBM samples. Grade I gliomas were used for comparison, and GAPDH was served as the endogenous control. *P* values were determined by Kruskal–Wallis One-Way ANOVA and Dunn’s multiple comparisons. *p < 0.05; **p < 0.01. **F** Representative immunoblots of LanCL2 and EGFR protein expression in newly diagnosed and relapsing GBM specimens. **G** Person’s correlation analysis showed that protein expression of LanCL2 and EGFR was not significantly correlated. **H** Chi-square test showed the correlations between the protein expression of LanCL2 & EGFR and *IDH1/2* mutation, *MGMT* methylation and *TERT* promoter mutation. **I**, **J** Protein overexpression of LanCL2 and EGFR was not significantly correlated with OS of GBM patients

**Fig. 5:**
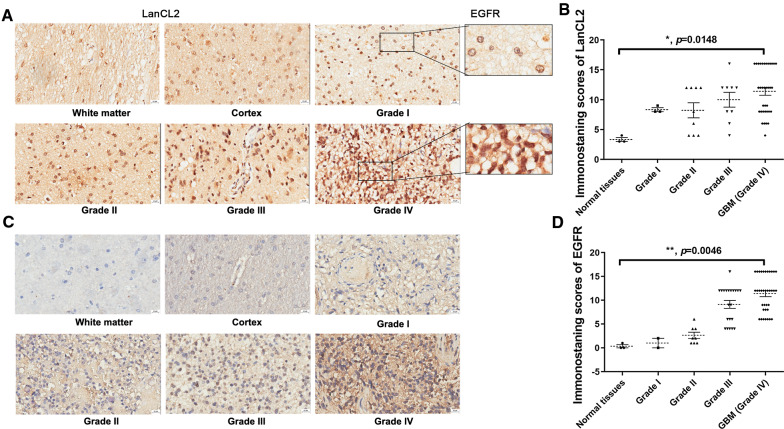
Protein expression and intracellular localization of LanCL2 was correlated with the grade of gliomas. **A** Immunohistochemistry analysis of LanCL2 in representative sections of grade I to IV gliomas. Sections of white matter and cortex were used for comparison. Bar = 20 μm. **B** IHC staining scores of LanCL2 in tumor sections. **C** Immunohistochemistry analysis of EGFR in representative sections of grade I to IV gliomas. Bar = 20 μm. **D** IHC staining scores of EGFR in tumor sections. *P* values were determined by Kruskal–Wallis One-Way ANOVA and Dunn’s multiple comparisons. *p < 0.05; **p < 0.01

To investigate the protein expression profiles of LanCL2 and EGFR, 72 GBM samples and 4 low-grade (grade I) glioma samples from our tumor banks were used. Compared with the grade I glioma control, the log_2_ relative protein expression values larger than 2 was regarded as overexpression. We found that overexpression of LanCL2 and EGFR was found in 38.89% and 58.33% of the total GBM samples (Fig. 4A, Additional file 1: Table S6). The protein expression of EGFR was markedly increased in GBM samples, whereas the expression levels of LanCL2 had no significant change (Fig. 4B, C). Interestingly, overexpression of LanCL2 was observed in relapsing GBM compared with newly diagnosed GBM (Fig. 4D, F). On the other hand, although both the newly diagnosed and relapsing GBM samples displayed elevated EGFR expression compared with the grade I glioma samples, no significant change was found between the newly diagnosed and relapsing GBM samples (Fig. 4E, F). Pearson’s correlation analysis showed that the expression levels of LanCL2 and EGFR were not correlated (Fig. 4G). Chi-square tests showed that overexpression of LanCL2 or EGFR was not significantly associated with *IDH1* or *TERT* mutations, and *MGMT* methylation (Fig. 4H). No significant association was also found between the expression of LanCL2/EGFR and OS of GBM patients (Figs. 3I, 4I, J). Subsequently, we used tissue microarray to investigate the expression pattern of LanCL2 and EGFR in GBM cells. Results also showed that the expression scores of both LanCL2 and EGFR were markedly increased in GBM tissues, compared with normal brain tissues (Fig. 5B, D). LanCL2 was expressed in both the normal brain tissues and gliomas. The protein expression level and intracellular localization of LanCL2 were correlated with the grade of gliomas. The higher the glioma grade, the higher the expression intensity of LanCL2. LanCL2 was mainly found in the nucleus and cytoplasm of high-grade glioma cells (grade III–IV), whereas it was expressed on the nuclear membrane of low-grade (grade I–II) glioma cells (Fig. 5A). On the other hand, EGFR was barely expressed in the normal brain tissues and low-grade gliomas, but was overexpressed in the grade III–IV gliomas. It was mainly located in the plasma membrane and cytoplasm of both low-grade and high-grade glioma cells (Fig. 5C).

## Discussion

Amplification and overexpression of *EGFR* are frequently occurred and extensively studied in GBM. Eley et al. found that approximate 34% (40 of 118 cases) of GBM samples contained *EGFR* amplification, and 50% (20 of 40 cases) of *EGFR*-amplified GBM samples displayed *LANCL2* co-amplification [19]. Concomitant amplification or copy number gain of two genes is a common phenomenon in cancers, such as *MYCN* and *DDX1* in neuroblastoma*, ERBB2/HER2* and *TOPOIIα* in prostate cancer [27–29]. Similar as *LANCL2* and *EGFR*, these genes are located in the same amplification region, which is the driving factor of their co-amplification. Since the co-amplification of *LANCL2* and *EGFR* was found in GBM in 2002, studies of *LANCL2* are barely reported in glioma till now. Only one study using integrative radiogenomic analysis found that the copy number and gene expression of *LANCL2* were significantly increased in multicentric GBM [27]. In this study, we firstly analyzed the TCGA database and found that the amplification frequencies of *LANCL2* and *EGFR* in GBM were the highest among 32 different types of tumors, indicating the high specificity of *LANCL2* and *EGFR* amplification in GBM. The frequencies of *LANCL2* and *EGFR* amplification in 575 GBM patients were approximate 28% and 44% respectively, and 62% of GBM samples with *EGFR* amplification contained *LANCL2* co-amplification, which were higher than the frequencies reported in Eley’s study. In addition, the amplification frequencies of *LANCL2* and *EGFR* in GBM were six to nine times higher than those in grade II-III gliomas. These findings were verified in 100 GBM samples of our tumor banks, which showed higher amplification and co-amplification frequencies of *LANCL2* and *EGFR* than the results analyzed in TCGA database. Moreover, *EGFR* is the top gene with the highest amplification frequency in the TCGA database of Glioblastoma Multiforme (PanCancer Atlas) (data not shown). These suggest that amplification of *EGFR* or co-amplification of *LANCL2* and *EGFR* were potential diagnostic markers for GBM patients.

Univariate analysis of TCGA database and our tumor banks showed that amplification of *LANCL2* or *EGFR*, and their co-amplification were significantly correlated with poor OS, but not PFS of GBM patients. However, since age and gender were significant variables associated with OS in GBM patients of TCGA database, multivariate analysis was performed. No prognostic value of amplification or co-amplification of *LANCL2* & *EGFR* for OS was shown in multivariate analysis, suggesting that interaction effects among these variables (age, gender, ethnicity, *LANCL2* and *EGFR* genes status) were significant. However, probably due to a smaller sample size of GBM patients, age and gender had no significant impact on the OS of GBM patients from our tumor banks, leading that *LANCL2* amplification was a significant independent prognostic factor for OS in multivariate analysis. In order to eliminate the interference of age, we divided GBM patients into two groups: younger (< 60 years) and older (≥ 60 years) patients. Multivariate analysis of samples from both TCGA database and our tumor banks demonstrated that *LANCL2* amplification was a significant independent prognostic factor for OS in younger GBM patients.

Subsequently, we found that *IDH1/2* mutation, but not *MGMT* methylation status was correlated with CNVs of *LANCL2* and *EGFR*. However, amplification of *LANCL2/EGFR* and their co-amplification were not associated with the OS and PFS of *IDH1/2*-wild-type GBM patients. Similar studies also find that *EGFR* amplification is closely associated with wild-type *IDH1/2* [28]. *CDKN2A/B* deletion, but not *TERT* mutation or *EGFR* amplification, was associated with worse OS and PFS of *IDH*-wild-type GBM patients [29]. Our findings indicated that CNVs of *LANCL2* and *EGFR* were not the independent prognostic factors for *IDH1/2*-wild-type GBM patients.

Numerous studies showed that both the mRNA and protein overexpression of EGFR, which were highly correlated with *EGFR* amplification, were the signatures and prognostic predictors for GBM patients [30–33]. However, paradox was delineated that the mRNA expression of *EGFR*, not protein expression, showed a close correlation with *EGFR* amplification [34]. Currently, no study has yet reported the expression profiles and prognostic values of LanCL2 in GBM. In this study, we found that the mRNA expression levels of *LANCL2* and *EGFR* were positively correlated in GBM samples of TCGA database. *LANCL2* mRNA expression was significantly increased in *LANCL2*-amplified samples, so was EGFR. In our tumor banks, we found that the protein expression of EGFR was elevated in GBM samples, whereas LanCL2 expression did not significantly change. The protein expression of LanCL2 and EGFR was not correlated with each other. On the other hand, mRNA and protein overexpression of LanCL2 or EGFR were not associated with OS and PFS in historical GBM patients of TCGA database and our tumor banks, let alone in *IDH1/2*-wild-type GBM patients. Nevertheless, the roles of LanCL2 and EGFR in GBM cells are of importance and can’t be ignored. Plenty of studies showed that EGFR and its active mutant EGFRvIII played critical roles in tumorigenesis, proliferation, angiogenesis, and invasion of GBM [35–38]. However, the role of LanCL2 in GBM remains elusive. In this study, LanCL2 protein overexpression was only found in relapsing GBM compared with newly diagnosed GBM, indicating that LanCL2 overexpression may be correlated with GBM recurrence. In contrast, no significant difference of EGFR expression was found between newly diagnosed and relapsing GBM. Moreover, no significant correlation between LanCL2 and EGFR protein expression was showed. These findings suggest the expression pattern and role of LanCL2 in GBM are independent to EGFR. A study demonstrated that the N-terminus of LanCL2 protein could be myristoylated and LanCL2 was located in the plasma membrane, juxta-nuclear vesicles, and the nucleus [39]. Here, our immunohistochemical result found that the expression and localization of LanCL2 was correlated with the grade of gliomas. The major localization of LanCL2 in GBM cells was in the nucleus and cytoplasm, whereas it was mainly expressed on the nuclear membrane of LGG cells. Another study showed that LanCL2 is a non-transmembrane G protein-coupled receptor, and its nuclear enrichment was induced by ABA or its unmyristoylation to activate ABA signaling [40]. Therefore, we speculated that LanCL2 maintains inactive in the cytoplasm of LGG cells, while LanCL2 transforms to the active form in GBM cells and then translocates into the nucleus.

## Conclusion

In summary, this study showed that amplification and mRNA overexpression of *LANCL2* and *EGFR*, and their co-amplification and co-expression frequently occurred in GBM patients, compared with patients with LGG. Multivariate analysis showed that *LANCL2* amplification was significantly correlated with reduced OS in younger (< 60 yrs) glioblastoma patients of TCGA database and our tumor banks. *LANCL2* or *EGFR* amplification, and their co-amplification were not associated with OS of older (≥ 60 yrs) or *IDH1/2*-wild-type GBM patients. In addition, mRNA and protein expression of LanCL2 and EGFR were not correlated with the prognosis of GBM patients. Taken together, amplification of *LANCL2* and *EGFR* were the independent diagnostic biomarkers for glioblastoma patients, and *LANCL2* amplification was a significant prognostic factor for OS in younger glioblastoma patients. The protein expression pattern and role of LanCL2 in GBM were independent to EGFR.

## Supplementary Information

**Additional file 1: Figure S1.** Prognostic values of *LANCL2* and *EGFR* amplification for OS and PFS in GBM patients of TCGA database. **Figure S2.** mRNA overexpression of LANCL2 and EGFR was not associated with prognosis of historical or *IDH1/2*-wild-type GBM patients. **Figure S3.** The prognostic values of amplification of *LANCL2* or *EGFR*, and their co-amplification in *IDH1/2*-wild-type GBM patients from Shenzhen Second People’s Hospital and Sun Yat-sen University Cancer Center. **Table S1.** Multivariate analysis by the Cox proportional hazard regression model in a forward manner in older GBM patients (age ≥ 60 yrs) of TCGA database. **Table S2.** Univariate analysis for OS and PFS in *IDH1/2*-wild-type GBM patients of TCGA database. **Table S3.** Multivariate analysis by the Cox proportional hazard regression model for OS in *IDH1/2*-wild-type GBM patients of TCGA database. **Table S4.** Multivariate analysis by the Cox proportional hazard regression model in a forward manner in older GBM patients (age ≥ 60 yrs) from tumor banks. **Table S5.** Amplification and co-amplification of *LANCL2* and *EGFR* in GBM samples of tumor banks. **Table S6.** Protein overexpression of LanCL2 and EGFR in GBM samples of tumor banks.

## Data Availability

The datasets analyzed during the current study are available in the TCGA repository, http://www.cbioportal.org.

## Abbreviations

ABA: Abscisic acid
CNV: Copy number variation
EGFR: Epidermal growth factor receptor
GBM: Glioblastoma multiforme
HR: Hazard ratio
IDH: Isocitrate dehydrogenase
IHC: Immunohistochemistry
LANCL2: Lanthionine synthetase C-like 2
LGG: Low-grade glioma
MGMT: O(6)-methylguanine-DNA methyltransferase
OS: Overall survival
PFS: Progression-free survival
RTK: Receptor tyrosine kinase
TASP: Testis adriamycin sensitivity protein
TERT: Telomerase reverse transcriptase
TCGA: The Cancer Genome Atlas
WHO: World Health Organization
