# Supplementary material for: Identification of prognostic values defined by copy number variation, mRNA and protein expression of LANCL2 and EGFR in glioblastoma patients
Source: J Transl Med. 2021 Aug 30;19:372. doi: 10.1186/s12967-021-02979-z (PMC8404333; doi:10.1186/s12967-021-02979-z)
Supplement: Supplementary file 1 — Additional file 1: Figure S1. Prognostic values of LANCL2 and EGFR amplification for OS and PFS in GBM patients of TCGA database. Figure S2. mRNA overexpression of LANCL2 and EGFR was not associated with prognosis of historical or IDH1/2-wild-type GBM patients. Figure S3. The prognostic values of amplification of LANCL2 or EGFR, and their co-amplification in IDH1/2-wild-type GBM patients from Shenzhen Second People’s Hospital and Sun Yat-sen University Cancer Center. Table S1. Multivariate analysis by the Cox proportional hazard regression model in a forward manner in older GBM patients (age ≥ 60 yrs) of TCGA database. Table S2. Univariate analysis for OS and PFS in IDH1/2-wild-type GBM patients of TCGA database. Table S3. Multivariate analysis by the Cox proportional hazard regression model for OS in IDH1/2-wild-type GBM patients of TCGA database. Table S4. Multivariate analysis by the Cox proportional hazard regression model in a forward manner in older GBM patients (age ≥ 60 yrs) from tumor banks. Table S5. Amplification and co-amplification of LANCL2 and EGFR in GBM samples of tumor banks. Table S6. Protein overexpression of LanCL2 and EGFR in GBM samples of tumor banks. [file 12967_2021_2979_MOESM1_ESM.docx]

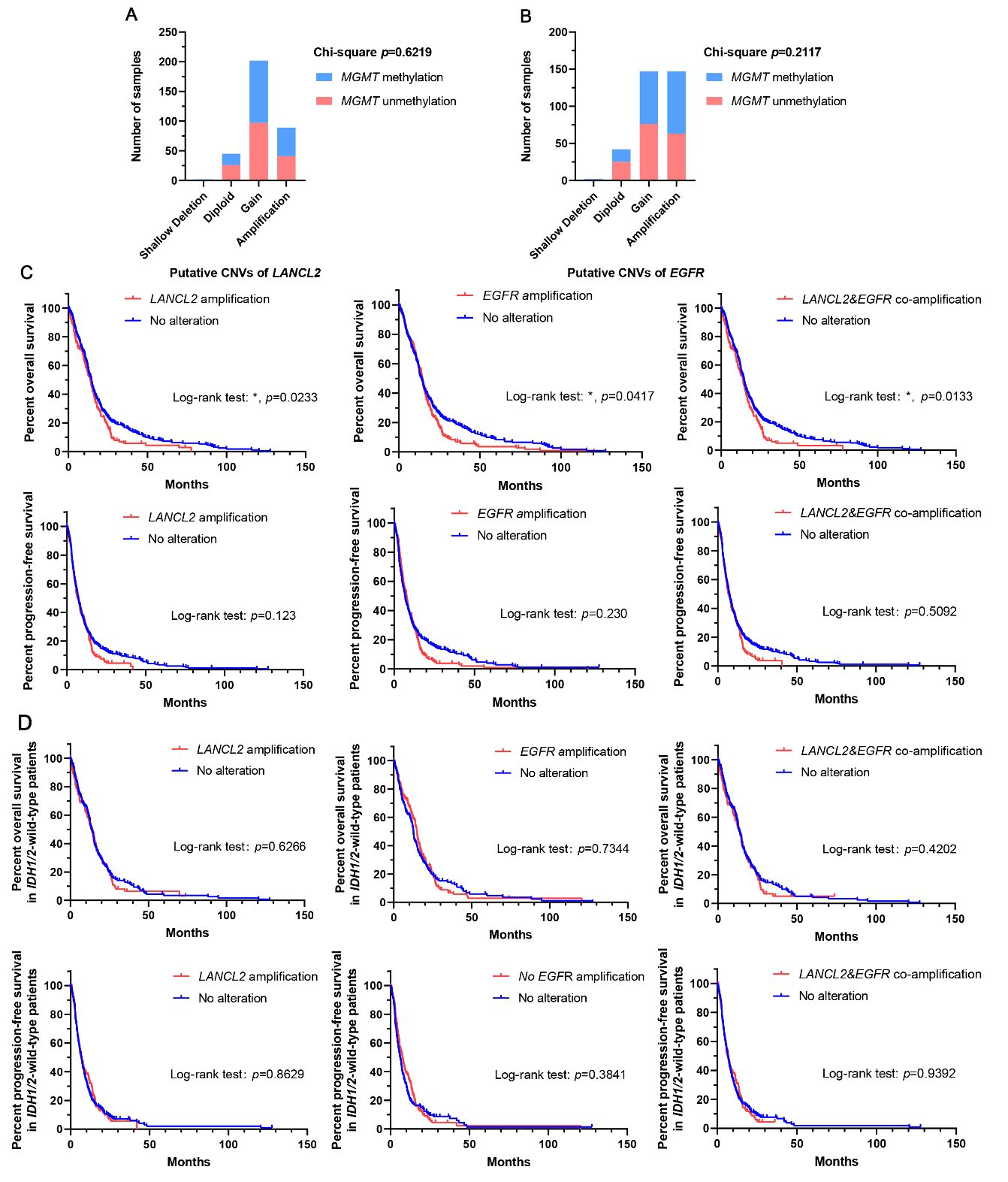


**Figure S1: Prognostic values of *LANCL2* and *EGFR* amplification for OS and PFS in GBM patients of TCGA database.** (A-B) Chi-square test of correlations between the amplification of *LANCL2* & *EGFR* and *MGMT* methylation in GBM patients. (C-D) Kaplan-Meier survival curves (log-rank test) of OS and PFS according to the amplification status of *LANCL2* and *EGFR* in historical (n=579) or *IDH1/2*-wild-type (n=353) GBM patients.


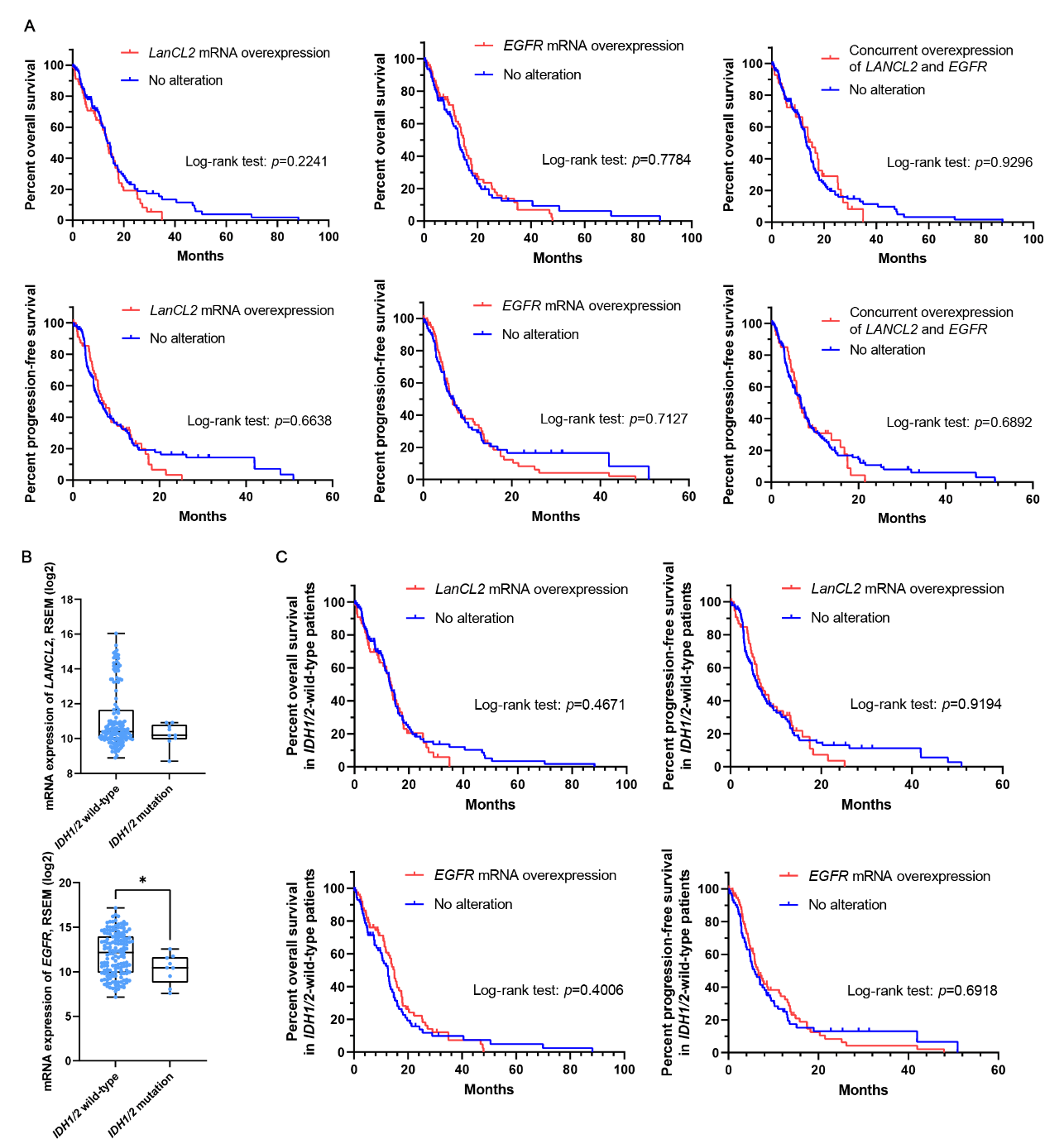


**Figure S2：mRNA overexpression of LANCL2 and EGFR was not associated with prognosis of historical or *IDH1/2*-wild-type GBM patients.** (A) Kaplan-Meier survival analysis showed that mRNA overexpression of *LANCL2* or *EGFR* was not associated with OS and PFS of GBM patients (n=154). (B) The correlations between mRNA expression of *LANCL2* or *EGFR* and *IDH1/2* mutation status. *P* values were determined by Mann-Whitney U test. *: *p* < 0.05. (C) Kaplan-Meier survival analysis showed that mRNA overexpression of *LANCL2* or *EGFR* was not associated with OS and PFS of *IDH1/2*-wild-type GBM patients (n=145).

**
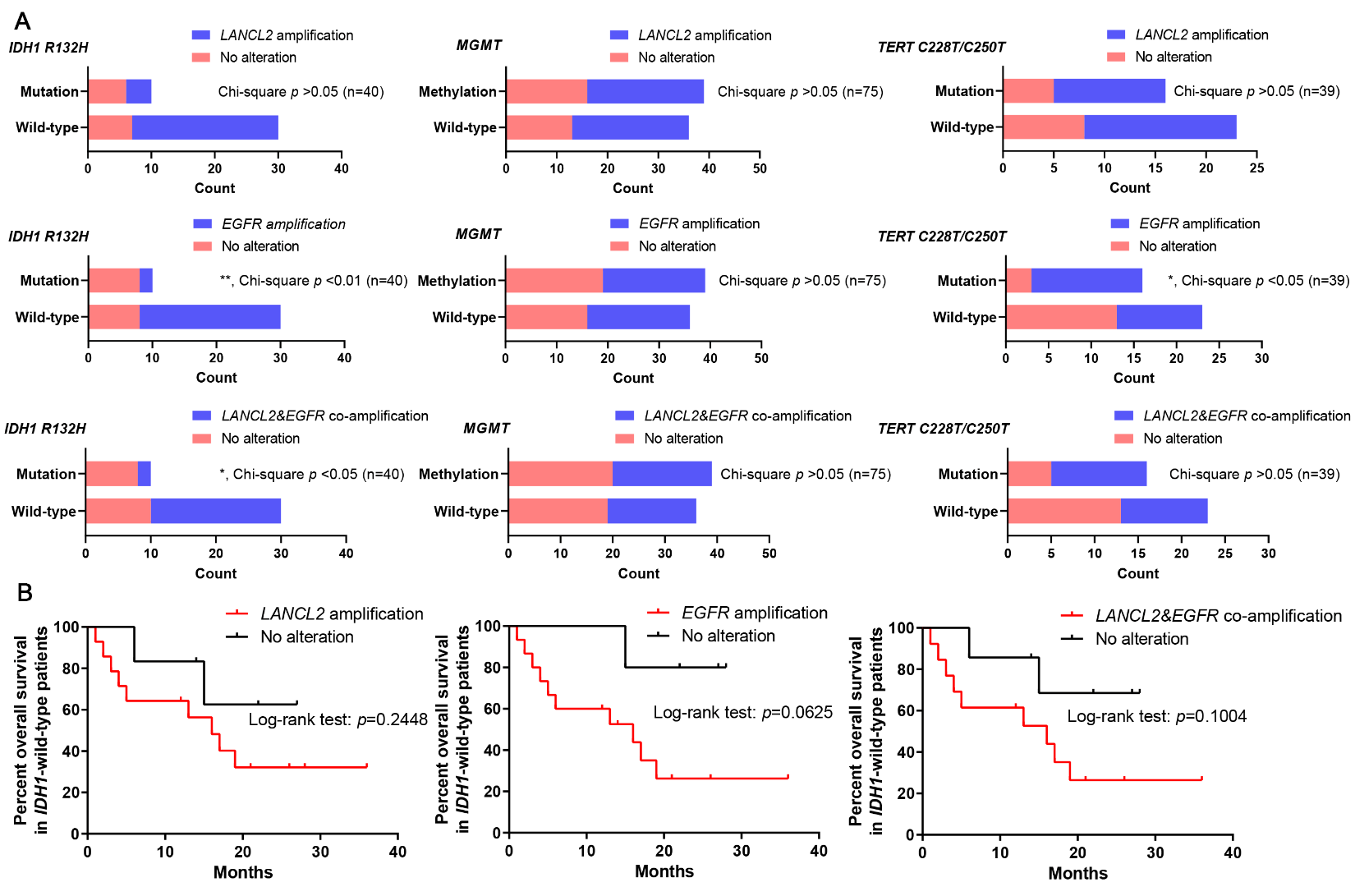
**

**Figure S3：**The prognostic values of amplification of *LANCL2* or *EGFR*, and their co-amplification in *IDH1/2*-wild-type GBM patients from Shenzhen Second People’s Hospital and Sun Yat-sen University Cancer Center. (A) Chi-square tests showed the correlations between the amplification or co-amplification of *LANCL2* & *EGFR* and *IDH1/2* mutation, *MGMT* methylation and *TERT* promoter mutation. (B) Kaplan-Meier survival analysis of *LANCL2* or *EGFR* amplification, and their co-amplification for OS in *IDH1/2*-wild-type GBM patients

**Table S1. Multivariate analysis by the Cox proportional hazard regression model in a forward manner in older GBM patients (age ≥60 yrs) of TCGA database.**

| **Variable** | **HR (95% CI)** | ***P*** |
| --- | --- | --- |
| Gender |  |  |
| Male vs Female | NA | 0.604 |
| LANCL2 gene status |  |  |
| Amplification vs No alteration | NA | 0.322 |
| EGFR gene status |  |  |
| Amplification vs No alteration | NA | 0.477 |
| LANCL2 & EGFR genes status |  |  |
| Co-amplification vs No alteration | NA | 0.322 |

HR, hazard ratio; CI, confidence interval; NA, not applicable.

**Table S2. Univariate analysis for OS and PFS in *IDH1/2*-wild-type GBM patients of TCGA database.**

| **Variable** | **No.(%)** | **Median OS (months)** | ***P*** | **Median PFS (months)** | ***P*** |
| --- | --- | --- | --- | --- | --- |
| Age (yrs) |  |  | <0.001 |  | 0.136 |
| ≥60 | 137 (57.56) | 11.28 |  | 5.92 |  |
| <60 | 101 (42.44) | 17.19 |  | 7.59 |  |
| Gender |  |  | 0.008 |  | 0.069 |
| Male | 141 (59.24) | 12.76 |  | 6.84 |  |
| Female | 97 (40.76) | 15.12 |  | 7.36 |  |
| Ethnicity |  |  | 0.753 |  | 0.121 |
| Hispanic or Latino | 4 (2.12) | 7.36 |  | 4.14 |  |
| Not Hispanic or Latino | 185 (97.88) | 12.95 |  | 6.84 |  |
| LANCL2 gene status |  |  | 0.627 |  | 0.863 |
| Amplification | 114 (32.29) | 13.61 |  | 6.84 |  |
| No alteration | 239 (67.71) | 13.78 |  | 6.41 |  |
| EGFR gene status |  |  | 0.734 |  | 0.384 |
| Amplification | 179 (50.71) | 12.62 |  | 6.02 |  |
| No alteration | 174 (49.29) | 14.93 |  | 7.63 |  |
| LANCL2 & EGFR genes status |  |  | 0.420 |  | 0.939 |
| Co-amplification | 113 (32.01) | 13.61 |  | 6.84 |  |
| No alteration | 240 (67.99) | 13.78 |  | 6.41 |  |

**Table S3. Multivariate analysis by the Cox proportional hazard regression model for OS in *IDH1/2*-wild-type GBM patients of TCGA database.**

| **Variable** | **HR (95% CI)** | ***P*** |
| --- | --- | --- |
| Age (yrs) |  |  |
| ≥60 vs <60 | 1.900 (1.389-2.598) | <0.001 |
| Gender |  |  |
| Male vs Female | 1.442 (1.049-1.983) | 0.024 |
| LANCL2 gene status |  |  |
| Amplification vs No alteration | NA | 0.907 |
| EGFR gene status |  |  |
| Amplification vs No alteration | NA | 0.242 |
| LANCL2 & EGFR genes status |  |  |
| Co-amplification vs No alteration | NA | 0.907 |

HR, hazard ratio; CI, confidence interval; NA, not applicable.

**Table S4. Multivariate analysis by the Cox proportional hazard regression model in a forward manner in older GBM patients (age ≥60 yrs) from tumor banks.**

| **Variable** | **HR (95% CI)** | ***P*** |
| --- | --- | --- |
| Gender |  |  |
| Male vs Female | NA | 0.963 |
| LANCL2 gene status |  |  |
| Amplification vs No alteration | NA | 0.454 |
| EGFR gene status |  |  |
| Amplification vs No alteration | NA | 0.503 |
| LANCL2 & EGFR genes status |  |  |
| Co-amplification vs No alteration | NA | 0.433 |

HR, hazard ratio; CI, confidence interval; NA, not applicable.

**Table S5. Amplification and co-amplification of *LANCL2* and *EGFR* in GBM samples of tumor banks.**

| **Affiliation** | **NO. of cases *LANCL2* amplification** | **NO. of cases *EGFR* amplification** | **NO. of cases co-amplification** | **Total cases** | **Percent *LANCL2* amplification** | **Percent *EGFR* amplification** | **Percent co-amplification** | **Percent co-amplification in *EGFR* amplification** |
| --- | --- | --- | --- | --- | --- | --- | --- | --- |
| Shenzhen Second People’s Hospital | 33 | 30 | 27 | 51 | 64.71% | 58.82% | 52.94% | 90.00% |
| Sun Yat-sen University Cancer Center | 29 | 25 | 20 | 49 | 59.18% | 51.02% | 40.82% | 80.00% |
| **Total** | 62 | 55 | 47 | 100 | 62.00% | 55.00% | 47.00% | 85.45% |

**Table S6. Protein overexpression of LanCL2 and EGFR in GBM samples of tumor banks.**

| **Affiliation** | **NO. of cases LanCL2 overexpression** | **NO. of cases EGFR overexpression** | **Total cases** | **Percent LanCL2 overexpression** | **Percent EGFR overexpression** |
| --- | --- | --- | --- | --- | --- |
| Shenzhen Second People’s Hospital | 19 | 27 | 47 | 40.43% | 57.45% |
| Sun Yat-sen University Cancer Center | 9 | 15 | 25 | 36.00% | 60.00% |
| **Total** | 28 | 42 | 72 | 38.89% | 58.33% |
